# Urinary nicotine metabolites are associated with cognitive impairment among the elderly in southern China

**DOI:** 10.18332/tid/170423

**Published:** 2023-10-04

**Authors:** Chao Huang, Xiaohu Ren, Benhong Xu, Peiyi Liu, Tian Li, Qinqin Zhu, Jia Huang, Xiao Chen, Desheng Wu, Xifei Yang, Feiqi Zhu, Jianjun Liu

**Affiliations:** 1 Shenzhen Key Laboratory of Modern Toxicology, Shenzhen Medical Key Discipline of Health Toxicology (2020-2024), Shenzhen Center for Disease Control and Prevention, Shenzhen, People’s Republic of China; 2 Cognitive Impairment Ward of Neurology Department, The Third Affiliated Hospital of Shenzhen University Medical College, Shenzhen, People’s Republic of China

**Keywords:** environmental tobacco smoke exposure, nicotine metabolites, mini-mental state examination, dose-response relationship, cross-sectional study

## Abstract

**INTRODUCTION:**

This study comprehensively assessed the association between eight metabolites of urinary nicotine and cognitive impairment.

**METHODS:**

This cross-sectional study was based on the data of Shenzhen Aging Related Disorder Cohort (SADC), including 51 elderly community data variables such as demographic characteristics, neuropsychological assessment and environmental factors, from July 2017 to November 2018. Participant’s cognitive function was assessed by Mini-Mental State Examination (MMSE) scale and urinary nicotine metabolite [including cotinine N-β-D-glucuronide (CotGluc), rac 4-hydroxy-4-(3-pyridyl) butanoic acid dicyclohexylamine salt (HyPyBut), trans-3’-hydroxy cotinine O-β-D-glucuronide (OHCotGluc), and cotinine (Cot), etc.] concentrations were measured by high-performance liquid chromatography-tandem mass spectrometry (LC-MS/MS). Generalized linear models and restricted cubic spline models were used to explore the relationships between the urinary levels of nicotine metabolite and cognitive function.

**RESULTS:**

A total of 296 individuals aged >60 years were included. Individuals in the third quartile of CotGluc had a 0.786 point (95% CI: -1.244 – -0.329) decrease or in the highest quartile of OHCotGluc had a 0.804 point (95% CI: -1.330 – -0.278) decreased in attention and calculation compared to those in the lowest quartile (all p for trend <0.05). Compared with those in the lowest quartile, individuals in the highest quartile of CotGluc, HyPyBut, OHCotGluc and Cot, respectively, corresponded to a 1.043 point (95% CI: -2.269–0.182), 1.101 points (95% CI: -2.391–0.188), 2.318 points (95% CI: -3.615 – -1.020), and 1.460 points (95% CI: -2.726 – -0.194) decreased in MMSE total score (all p for trend <0.05). A non-linear dose-response relationship between urinary levels of CotGluc, HyPyBut, OHCotGluc or Cot and cognitive function (all overall p<0.05, non-linear p<0.05). Subgroup analysis showed that urinary levels of CotGluc, OHCotGluc or Cot were significantly negatively associated with cognitive function (all p for trend <0.05) among females and non-smokers.

**CONCLUSIONS:**

The findings highlight the public health implications of environmental tobacco smoke exposure, and effective interventions need to be performed for vulnerable populations.

## INTRODUCTION

Tobacco smoke exposure can lead to damaged health and even death of millions of people every year, with over 1 billion current smokers in the world consuming 7.41 trillion cigarette-equivalent of tobacco, causing 7.69 million deaths and 200 million disability-adjusted life-years in 2019^1,2^. Long-term smoking or exposure to environmental tobacco smoke elevates the hazards of respiratory, cardio-cerebrovascular, neurological, and other diseases^3^. Nicotine is the principal content alkaloid in tobacco. As a specific exposure marker of tobacco smoke, nicotine is absorbed through the respiratory tract and skin and metabolized into a variety of products through the liver CYP2A6 enzyme^4^. At present, urinary nicotine metabolites have been detected, including cotinine, trans-3’-hydroxycotinine, nicotine-N-oxide, cotinine-N-oxide, nor-nicotine, nor-cotinine, and 4-hydroxy-4-(3-pyridy1) butyric acid. Cotinine can reflect the exposure hazards of tobacco smoke (3–4 days) due to the long biological half-life (18–24 h)^5^. Therefore, the detection of cotinine concentration in biological samples such as blood, urine and saliva is the most common method to quantify smoking or environmental tobacco smoke exposure^6^.

The neurotoxicity assessment of tobacco exposure is limited by only the analysis of one urinary nicotine metabolite. Cotinine barely accounts for 10%–15% of the total nicotine excretion^4^, and the concentration is affected by multiple factors such as genetics, gender, and nicotine metabolism rate^7^. It is necessary to comprehensively assess the total nicotine exposure of the body by detecting urinary multiple nicotine metabolites^8,9^. To explore the association between urinary nicotine metabolites and cognitive function, high-performance liquid chromatography-tandem mass spectrometry (LC-MS/MS) was used to detect eight urinary nicotine metabolites [including cotinine N-β-D-glucuronide (CotGluc), rac 4-hydroxy-4-(3-pyridyl)butanoic acid dicyclohexylamine salt (HyPyBut), trans-3’-hydroxy cotinine O-β-D-glucuronide (OHCotGluc), (S)-cotinine N-oxide (CNO), nicotine-N-β-glucuronide (NicGluc), (1’S, 2’S)-nicotine-1’-oxide (NNO), trans-3’-hydroxy cotinine (OHCot), cotinine (Cot)] and Mini-Mental State Examination (MMSE) scale was used to assess cognitive function in the elderly from Shenzhen, China.

## METHODS

### Study population

This cross-sectional study is based on the Shenzhen Aging Related Disorder Cohort (SADC)^10^. SADC included 51 elderly community data variables such as demographic characteristics, lifestyle factors, neuropsychological assessment, genetic information and environmental factors, from July 2017 to November 2018. A total of 701 participants were assessed using the MMSE scale in this study. Firstly, we excluded 104 individuals with self-reported disease, including dementia (n=14), Parkinson’s disease (n=9), stroke (n=27), traumatic brain injury (n=32) and 22 individuals for psychotropic drug use. Thereafter, we excluded 301 individuals with missing data on education level (n=33), hypertension (n=21), hyperlipidemia (n=14), diabetes (n=25), smokers or passive smokers (n=41), alcohol consumption (n=27), noon break (n=17), kidney function or hepatic function index (n=5) as well as 118 individuals without data on urinary nicotine metabolites. The selection process is shown in the flowchart of Supplementary file Figure S1. Finally, 296 individuals were included in this study. All participants signed an informed consent, the research protocol was approved by the Medical Ethics Research Committee of Shenzhen Center for Disease Control and Prevention (Approval number: R2017001).

### Data collection

A face-to-face interview was performed by investigators using a questionnaire to collect data from the participants. The investigation contained the following items: general demographics, personal and family health histories, tobacco smoking and alcohol consumption. Some definitions in this study are as follows:

- Hypertension: systolic blood pressure ≥140 mmHg, diastolic blood pressure ≥90 mmHg, previously diagnosed hypertension patients or antihypertensive drug use;
- Hyperlipidemia: total cholesterol ≥5.18 mmol/L, triglyceride ≥1.7 mmol/L, low-density lipoprotein cholesterol ≥3.37 mmol/L, high-density lipoprotein cholesterol^11^ ≤1.0mmol/L or previously diagnosed hyperlipidemia or lipid-lowering drug use;
- Diabetes: fasting blood glucose concentration ≥7.0 mmol/L, previously diagnosed diabetes or hypoglycemic drug use;
- Smokers: smoked more than 1 cigarette/day in the past six months;
- Passive smokers: exposed to environmental tobacco smoke more than 1 time/week (≥15 min/time) in the past six months;
- Alcohol consumption: drank alcohol more than once a week for over six months; and
- Noon break: sleep at noon for about 0.5–1 h/day in the past six months.

### Cognitive function assessment

The MMSE scale was used to assess participants’ cognitive function under the guidance of neurologists. The scale covered five cognitive subdomains, including orientation (10 points), registration (3 points), attention and calculation (5 points), recall (3 points), and language and praxis (9 points), with a total of 30 points, with a lower score indicating worse cognitive function performance. In this study, the MMSE scale scores were regarded as continuous dependent variables.

### Laboratory measurements

Blood samples (5 mL) and urine samples (8 mL) were collected from all participants in the morning after an overnight fasting. The blood specimens were centrifuged at 3000 rpm for 5 min at room temperature to separate plasma and serum^10^ for biochemical analyses. An automatic biochemical analyzer (7600-010, Hitachi, Ltd., Tokyo, Japan) was used to detect kidney function (BUN: blood urea nitrogen; SUA: serum uric acid; SCr: serum creatinine) and hepatic function (TBil: total bilirubin; ALT: glutamic-pyruvic transaminase; AST: glutamic-oxaloacetic transaminase).

Concentrations of urinary nicotine and its metabolites (CotGluc, HyPyBut, OHCotGluc, CNO, NicGluc, NNO, OHCot, Cot) were measured by LC-MS/MS (1200 series/6410 Triple Quad LC/MS, Agilent Technologies Inc., Santa Clara, CA, USA) based on the method^12^ described in the Supplementary file, and shown in Supplementary file Table S1. The limits of detection (LOD) ranged 0.04–6.60 ng/mL for urinary concentrations of nicotine and its metabolites. Values of urinary nicotine and its metabolites below the LOQ were replaced by LOQ/2.

### Statistical analysis

One-Way ANOVA, Kruskal-Wallis H and chi-squared tests were correspondingly used to compare normally, non-normally continuous (including urinary nicotine metabolites concentrations) and categorical variables (including gender, education level, hypertension, hyperlipidemia, diabetes, smokers, passive smokers, alcohol consumption and noon break) between quartiles of MMSE score groups. Values of urinary nicotine metabolites were log10-transformed to approximately normal distributions before analysis. Spearman’s rank correlations coefficient was calculated between urinary nicotine metabolites concentrations and cognitive function (global and subdomains)^13^.

Generalized linear models (GLM) were used to explore associations between each nicotine metabolite’s level and cognitive function (global and subdomains); the participants were divided into four subgroups (i.e. Q1 as the reference group, Q2, Q3 and Q4) according to the quartile values of individual urinary nicotine metabolites. Associations were adjusted for gender, age, education level, hypertension, hyperlipidemia, diabetes, smokers, passive smokers, alcohol consumption, noon break, BUN, SUA, SCr, TBill, ALT, AST and urine creatinine. The median value of each nicotine metabolite’s concentration in each nicotine metabolite’s quartile (log10-transformed urinary metal value) was entered into GLM as a continuous variable for the trend test. Restricted cubic spline (RCS) models were constructed to analyze the dose-response relationship between a urinary nicotine metabolite’s level and cognitive function (global and subdomains)^8^. The knots were set to the 25th, 50th, and 75th percentiles of each urinary nicotine metabolite value, with the 50th percentile for each urinary nicotine metabolite as the reference value. All data were analyzed using R (version 4.1.2; R Foundation for Statistical Computing). Statistical significance was defined as p<0.05 (two-tailed)^13^.

## RESULTS

### Characteristics of participants

As shown in Table 1, of all the 296 participants, 51.4% were males and the mean age was 68.5 years. Compared to those with the lowest MMSE score, participants with a higher MMSE score were more likely to be male^14^, had higher education level, and had a lower concentration of BUN (all p<0.05). MMSE subdomain (orientation, registration, attention and calculation, recall, language and praxis) scores were all significantly higher in participants with higher MMSE score (all p<0.05)^14^. The median values of CotGluc, HyPyBut, OHCotGluc, CNO, NicGluc, NNO, OHCot and Cot for all participants were 1.07, 2.47, 2.80, 0.47, 0.26, 0.69, 2.70 and 0.72 ng/mL, respectively. The levels of CotGluc, HyPyBut, OHCotGluc, OHCot and Cot in individuals with MMSE score <14 were significantly higher than those with MMSE score >24 (all p<0.05).

**Table 1:** Baseline demographics and clinical characteristics of participants, Shenzhen, 2017–2018 (N=296)

*Characteristics*	*Total (N=296) n (%)*	*Quartiles of MMSE score*				*p*
		*<14 (N=75) n (%)*	*14–22 (N=84) n (%)*	*22–24 (N=105) n (%)*	*>24 (N=32) n (%)*	
Gender, male	152 (51.4)	27 (36.0)	48 (54.8)	61 (58.1)	18 (56.3)	**0.021^a^**
Age (years), mean ± SD	68.50 ± 6.21	68.20 ± 6.59	69.74 ± 6.96	68.03 ± 5.61	67.50 ± 4.74	0.176^b^
Education level (years)						**0.000^a^**
≤9	153 (51.7)	57 (76.0)	48 (57.1)	38 (36.2)	10 (31.3)	
>9–12	88 (29.7)	12 (16.0)	24 (28.6)	39 (37.1)	13 (40.6)	
>12	55 (18.6)	6 (8.0)	12 (14.3)	28 (26.7)	9 (28.1)	
Hypertension	107 (36.1)	31 (41.3)	25 (29.8)	39 (37.1)	12 (37.5)	0.488^a^
Hyperlipidemia	31 (10.5)	7 (9.3)	8 (9.5)	10 (9.5)	6 (18.8)	0.464^a^
Diabetes	56 (18.9)	14 (18.7)	18 (21.4)	20 (19.0)	4 (12.5)	0.750^a^
Smokers	72 (24.3)	17 (22.7)	24 (28.6)	25 (23.8)	6 (18.8)	0.690^a^
Passive smokers	27 (9.1)	10 (13.3)	9 (10.7)	4 (3.8)	4 (12.5)	0.115^a^
Alcohol consumption	48 (16.2)	12 (16.0)	13 (15.5)	18 (17.1)	5 (15.6)	0.995^a^
Noon break	223 (75.3)	54 (72.0)	66 (78.6)	78 (74.5)	25 (78.1)	0.779^a^
***Measurements***	***Mean ± SD***	***Mean ± SD***	***Mean ± SD***	***Mean ± SD***	***Mean ± SD***	***p***
BUN (mmol/L)	6.06 ± 2.01	6.35 ± 2.04	6.43 ± 2.12	5.63 ± 1.56	5.83 ± 2.63	**0.021^b^**
SUA (μmol/L)	391.94 ± 102.10	377.81 ± 108.89	395.47 ± 102.63	406.15 ± 104.73	369.15 ± 64.46	0.160^b^
SCr (μmol/L)	85.11 ± 32.81	82.03 ± 25.88	88.77 ± 45.31	84.39 ± 21.35	85.10 ± 39.49	0.627^b^
TBill (μmol/L)	15.60 ± 5.00	14.55 ± 4.36	15.87 ± 4.95	16.04 ± 5.60	15.91 ± 4.27	0.214^b^
ALT (U/L)	23.59 ± 30.48	29.00 ± 57.21	21.35 ± 12.39	21.69 ± 10.33	23.06 ± 12.67	0.358^b^
AST (U/L)	23.46 ± 25.24	28.39 ± 48.40	21.43 ± 6.47	22.03 ± 8.22	21.91 ± 7.15	0.278^b^
Urine creatinine (mmol/L)	9.75 ± 6.68	8.86 ± 5.10	9.49 ± 7.15	10.51 ± 7.17	10.03 ± 7.04	0.413^b^
**MMSE subdomains** (score)						
Orientation	8.84 ± 1.47	7.68 ± 2.12	8.95 ± 0.99	9.35 ± 0.78	9.59 ± 0.62	**0.000^b^**
Registration	2.74 ± 0.60	2.43 ± 0.89	2.81 ± 0.48	2.85 ± 0.41	2.94 ± 0.25	**0.000^b^**
Attention and calculation	2.94 ± 1.56	1.39 ± 1.35	2.63 ± 1.23	3.83 ± 0.97	4.44 ± 0.84	**0.000^b^**
Recall	0.61 ± 0.83	0.33 ± 0.70	0.50 ± 0.77	0.71 ± 0.79	1.22 ± 1.04	**0.000^b^**
Language and praxis	6.05 ± 1.84	4.21 ± 1.65	6.00 ± 1.45	6.78 ± 1.21	8.12 ± 0.91	**0.000^b^**
***Urinary nicotine metabolites (ng/mL)***	***Median (IQR)***	***Median (IQR)***	***Median (IQR)***	***Median (IQR)***	***Median (IQR)***	***p***
CotGluc	1.07 (0.02–8.12)	1.58 (0.02–18.06)	1.38 (0.02–19.08)	1.10 (0.02–8.70)	0.02 (0.02–0.02)	**0.000^c^**
HyPyBut	2.47 (0.05–10.01)	2.71 (0.93–2.71)	2.57 (0.05–11.67)	2.79 (0.05–8.40)	0.05 (0.05–3.49)	**0.013^c^**
OHCotGluc	2.80 (0.23–6.35)	4.67 (1.23–8.80)	3.55 (1.24–6.50)	2.21 (0.23–5.72)	0.23 (0.23–0.23	**0.000^c^**
CNO	0.47 (0.06–1.57)	0.51 (0.15–2.45)	0.34 (0.06–1.98)	0.37 (0.06–1.64)	0.88 (0.06–1.33)	0.807^c^
NicGluc	0.26 (0.02–2.11)	0.25 (0.02–1.58)	0.23 (0.02–3.23)	0.16 (0.02–1.84)	1.23 (0.14–3.42)	0.121^c^
NNO	0.69 (0.13–2.47)	0.72 (0.25–2.66)	0.48 (0.10–3.81)	0.60 (0.04–2.39)	1.18 (0.76–2.20)	0.078^c^
OHCot	2.70 (0.87–8.08)	3.50 (0.97–15.49)	3.43 (1.48–9.16)	2.25 (0.75–7.12)	0.28 (0.28–4.53)	**0.003^c^**
Cot	0.72 (0.04–3.08)	1.22 (0.11–4.29)	0.83 (0.04–5.67)	0.64 (0.04–2.04)	0.04 (0.04–0.04)	**0.000^c^**

MMSE: Mini-Mental State Examination. BUN: blood urea nitrogen. SUA: serum uric acid. SCr: serum creatinine. TBill: total bilirubin. ALT: glutamic-pyruvic transaminase. ASL: glutamic-oxaloacetic transaminase. CotGluc: cotinine N-β-D-glucuronide. HyPyBut: rac 4-hydroxy-4-(3-pyridyl) butanoic acid dicyclohexylamine salt. OHCotGluc: trans-3-’hydroxy cotinine O-β-D-glucuronide. CNO: (S)-cotinine N-oxide. NicGluc: nicotine-N-β-glucuronide. NNO: (1’S, 2’S)-nicotine-1’-oxide. OHCot: trans-3’-hydroxy cotinine. Cot: cotinine. SD: standard deviation. IQR: interquartile range.

a Chi-squared test compares the differences of categorical variables’ distribution between groups.

b One-Way ANOVA compares the differences between groups of means.

c Kruskal-Wallis H test is used for rank sum test of independent samples.

### Composition profiles of urinary nicotine metabolites

**Figure 1:**
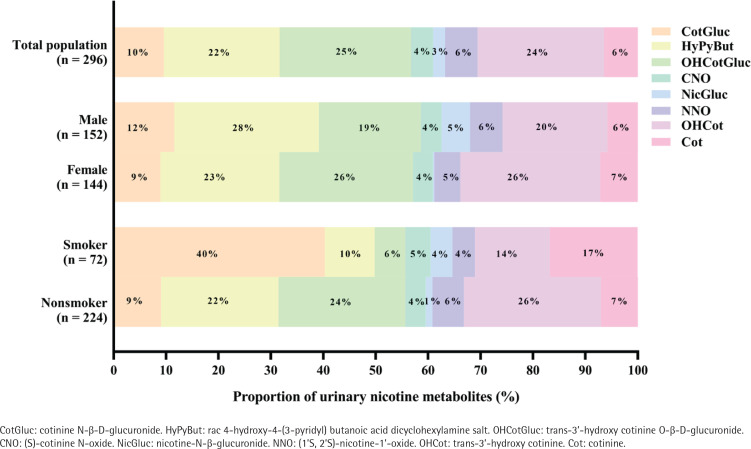
Compositional profiles of nicotine metabolites in urine samples, Shenzhen, 2017–2018

As shown in Figure 1, in the total population (n=296), the predominant compound was OHCotGluc, accounting for 25%, followed by OHCot (24%), HyPyBut (22%), and CotGluc (10%). In the male group (n=152), the top three urinary nicotine metabolites concentrations were: HyPyBut (28%) > OHCot (20%) > OHCotGluc (19%), and in the female group they were: OHCotGluc (26%) = OHCot (26%) > HyPyBut (23%). In the smoker group (n=72), the top three urinary nicotine metabolites concentrations were: CotGluc (40%) > Cot (17%) > OHCot (14%), however, OHCot (26%), OHCotGluc (24%) and HyPyBut (22%) were the top three nicotine metabolites in the non-smoker group (n=224).

### Correlations analysis of urinary nicotine metabolites and cognitive function

**Figure 2:**
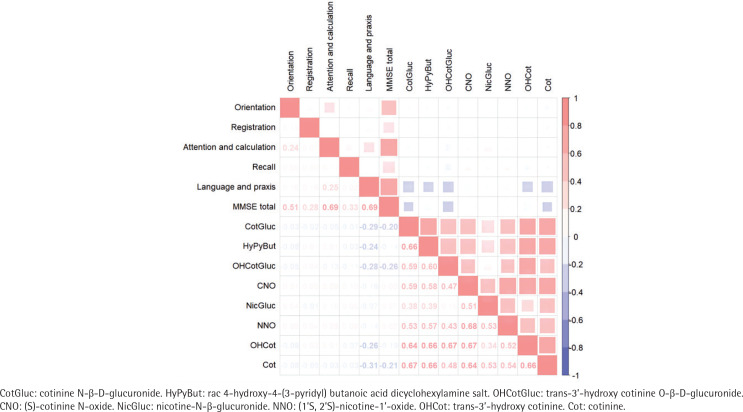
Heatmap for Spearman’s rank correlation coefficients between urinary nicotine metabolites concentrations and MMSE total and subdomain scores, Shenzhen, 2017–2018

As shown in Figure 2, Spearman correlation analysis revealed the positive correlations between the concentrations of eight nicotine metabolites with correlation coefficients ranging from 0.17 (OHCotGluc vs NicGluc) to 0.68 (CNO vs NNO) (all p<0.05). Strong correlations between attention and calculation or language and praxis with MMSE total score (both with a correlation coefficient = 0.69; all p<0.05). CotGluc, HyPyBut, OHCotGluc, OHCot and Cot exhibited slightly negative correlations with cognitive function (correlation coefficient ranging from -0.14 to -0.26, all p<0.05).

### Associations between urinary nicotine metabolites and cognitive function

**Figure 3:**
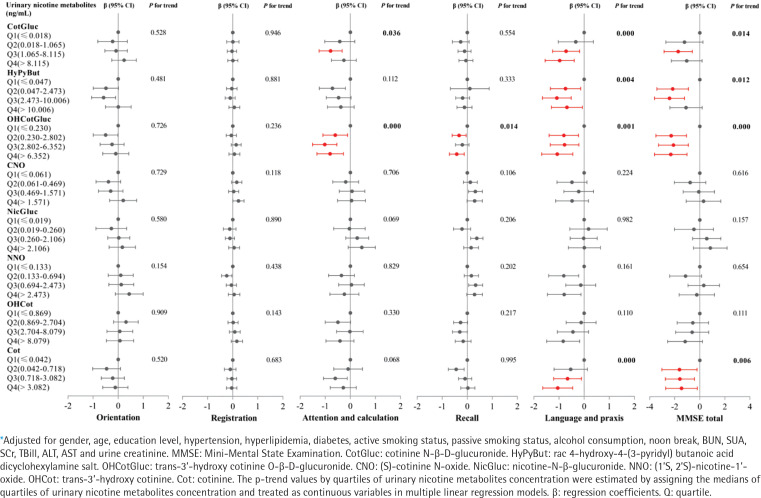
Generalized linear models were performed to assess associations* between urinary nicotine metabolites and cognitive function, Shenzhen, 2017–2018

As shown in Figure 3, GLM were used to explore the associations between urinary nicotine metabolites and cognitive function, and adjusted for potential confounders (gender, age, education level, hypertension, hyperlipidemia, diabetes, smokers, passive smokers, alcohol consumption, noon break, BUN, SUA, SCr, TBill, ALT, AST and urine creatinine). We found that individuals in the third quartile of CotGluc had a 0.786 point (95% CI: -1.244 – -0.329) decrease in attention and in the highest quartile of OHCotGluc had a 0.804 point (95% CI: -1.330 – -0.278) decrease in attention and calculation compared with those in the lowest quartile (all trend p<0.05). Compared with those in the lowest quartile, individuals in the highest quartile of CotGluc, HyPyBut, OHCotGluc and Cot, respectively, had a 0.974 point (95% CI: -1.553 – -0.396), 0.683 point (95% CI: -1.300 – -0.066), 1.070 point (95% CI: -1.693 – -0.447), and 1.051 point (95% CI: -1.650 – -0.453) decrease in language and praxis, or 1.043 point (95% CI: -2.269–0.182), 1.101 point (95% CI: -2.391–0.188), 2.318 point (95% CI: -3.615 – -1.020), and 1.460 point (95% CI: -2.726 – -0.194) decrease in MMSE total score (all trend p<0.05). As shown in Supplementary file Figure S2, after adjusting for the same confounders with GLM, the RCS models revealed a non-linear dose-response relationship between urinary levels of CotGluc, HyPyBut, OHCotGluc or Cot and cognitive function (all overall p<0.05, non-linear p<0.05).

### Subgroup analysis

**Figure 4:**
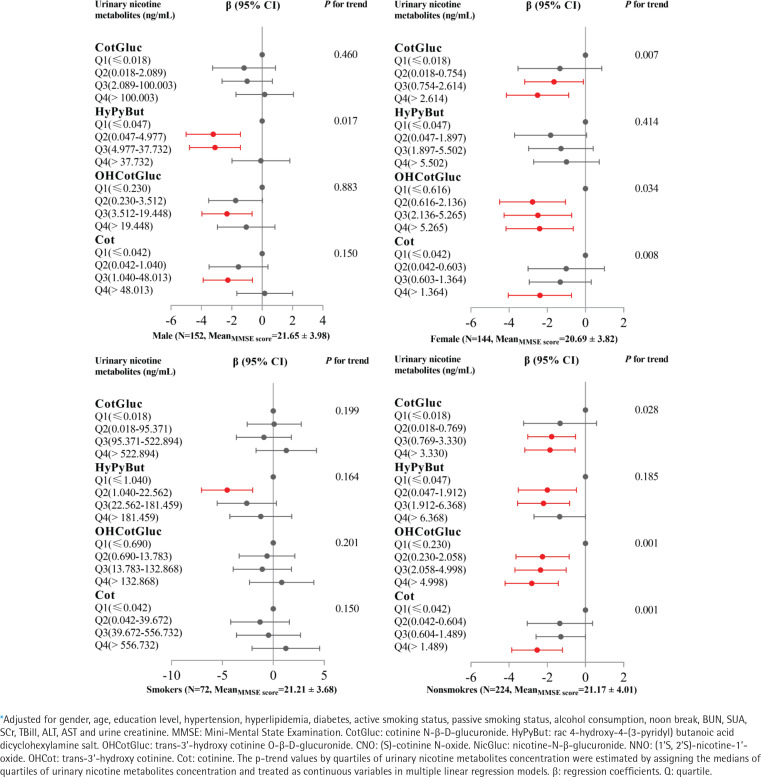
Generalized linear models were performed to assess associations* between urinary nicotine metabolites and cognitive function, by gender and smoking status, Shenzhen, 2017–2018

As shown in Supplementary file Table S2, MMSE total score in males was significantly higher than in females (p=0.034); however, there was no significant difference between smokers and non-smokers (p=0.949). Urinary levels of CotGluc, HyPyBut, OHCotGluc or Cot in males or smokers, were significantly higher than in females or non-smokers (all p<0.05). As shown in Figure 4, GLM results showed that urinary levels of CotGluc, OHCotGluc or Cot were significantly negatively associated with cognitive function (all trend p<0.05) in females or non-smokers. As shown in Supplementary file Figure S3, the RCS models revealed a non-linear dose-response relationship between urinary levels of CotGluc, HyPyBut, OHCotGluc or Cot and cognitive function in males (all overall p<0.05, non-linear p<0.05), however, a linear dose-response relationship between urinary levels of CotGluc, HyPyBut, OHCotGluc or Cot and cognitive function were found in females (all overall p<0.05, non-linear p>0.05). On the other hand, we found a linear dose-response relationship between urinary levels of CotGluc, OHCotGluc or Cot and cognitive function in non-smokers (all overall p<0.05, non-linear p>0.05), while there was a non-linear dose-response relationship between urinary HyPyBut levels and cognitive function (overall p<0.05, non-linear p=0.01).

## DISCUSSION

We found that urinary nicotine metabolites CotGluc, HyPyBut, OHCotGluc or Cot were associated with worse cognitive performance in attention and calculation or language and praxis among elderly from southern China, the associations were independent of potential confounders such as gender, age, hypertension, diabetes, smoking, alcohol consumption, kidney function, and hepatic function. Urinary nicotine metabolites are linked to declining cognitive performance among females and non-smokers. The findings highlight the public health implications of environmental tobacco smoke exposure, and effective interventions need to be performed for vulnerable populations.

Nicotine is a high-toxicity alkaloid that mainly exist in tobacco, tomatoes, eggplants, and other tobacco plants in the Solanaceae family. As an agonist of nicotinic acetylcholine receptors (nAChRs), nicotine has a higher affinity than acetylcholine (ACh), and can easily replace ACh to bind to nAChRs in the central and peripheral nervous systems^15,16^, resulting in a series of neurotoxic effects. Numerous studies have explored the link between tobacco smoke and cognitive impairment^17,18^, even among non-smokers^19-21^. The developmental neurotoxicity of nicotine may disrupt the balance of cholinergic transmission, and prenatal exposure to nicotine severely affects the neurodevelopment, cognition, and behavioral production of offspring^22-24^. Childhood tobacco smoke exposure can have sustained negative effects on cognitive function and brain structure^25,26^, and even affect cognitive ability in middle age^27^. Aging-related environmental tobacco smoke exposure is associated with poorer cognitive performance^28,29^. However, nicotine can improve cognitive performance in MCI patients and is also found in non-smokers^30-32^. These contrary results may have the following reasons: first, nicotine has multiple metabolites, which have different effects on cognitive ability. Recent studies reported that nicotine metabolites HyPyBut or NicGluc are positively associated with cognitive performance^12,29^. In this study, CNO and NicGluc showed a trend of cognitive protection, although not statistically significant. This may be due to differences in sample size, confounding factors adjustment, and cognitive function assessment tool. The opposite correlation between different metabolites of nicotine and cognitive function reflects the coexistence of neurotoxicity and neuroprotection in nicotine. Second, it is biased to assess the association with cognitive impairment by qualitative self-reporting smoking status. The neuroprotection effects of nicotine may be offset by other neurotoxic compounds in cigarette smoke, including heavy metals (such as arsenic), polycyclic aromatic hydrocarbons, hydrogen cyanide and other compounds. Finally, the effect of nicotine on cognitive function may depend on the patient’s baseline performance^33^, with a certain dose-dependent effect. These complex studies revealed the non-linear relationship of nicotine neurotoxicity.

This study has confirmed that nicotine metabolites are primarily associated with attention and calculation, as well as language and praxis. The China Health and Retirement Longitudinal Study (CHARLS) has shown that secondhand smoke exposure significantly reduced visual-spatial ability and situational memory function among elderly females^34^. Childhood tobacco smoke exposure has been linked to the impaired development of the cerebral cortex, frontal lobe, parietal lobe and temporal lobe^26^, resulting in poor attention, language acquisition and visual processing ability^25^. Animal experiments have shown that attention cognitive impairment caused by prenatal nicotine exposure can be improved by galantamine in mice^35^. In this regard, we can carry out cognitive recovery training in specific cognitive areas (attention, calculation, language and praxis). Cognitive training aims to improve cognitive function by utilizing the plasticity of the central nervous system. Cognitive training will enhance the overall and regional cerebral blood volume of the elderly, improve the connection in the central executive brain network, and may also increase the functional connection between the hippocampus and frontal and temporal lobe regions, thus promoting cognitive function^36^. On the other hand, it is also necessary to control smoking in public places and decrease environmental tobacco smoke exposure. Combining tobacco exposure reduction with specific cognitive rehabilitation training may provide a more effective strategy to preventing cognitive decline caused by tobacco smoke.

We noticed that the relationship between nicotine metabolites and cognitive function is stronger in females and non-smokers. The median concentration of cotinine in females (0.603 ng/mL) or non-smokers (0.604 ng/mL) is much lower than that in males (1.040 ng/mL) or smokers (39.672 ng/mL) in this study, even other nicotine metabolites. In addition, the sample size of smokers may lead to a lack of statistical efficacy in this association. Shenzhen vigorously promotes the construction of smoke-free cities, and people have high humanistic quality. Nevertheless, females and non-smokers are still high-risk groups exposed to environmental tobacco smoke. Accurate prevention and intervention targeting sensitive populations have important public health implications.

This study performed urinary nicotine and its metabolites^8^ to explore the relationship between tobacco smoke exposure and cognitive ability and subdomains, which can more comprehensively reflect environmental tobacco smoke exposure than self-reporting questionnaire surveys or single biomarkers. In addition, after considering a large number of potential confounding factors (such as gender, age, chronic illness, kidney function and hepatic function), we confirmed the non-linear relationship between nicotine metabolites and cognitive function and subdomains and further revealed that females and non-smokers are sensitive populations.

### Limitations

There are some limitations in this study. First, the relationship between high nicotine levels and cognitive impairment may be overlooked due to the small sample size of the smoking group. Second, the cognitive impairment of tobacco smoke may be affected by non-nicotine neurotoxic compounds. Third, the cross-sectional study restricted the ability to infer a causal relationship between them, and the results of elderly people in southern China cannot be extrapolated to other regions and populations. Finally, MMSE is a screening tool for cognitive improvement but does not substitute a full neurological evaluation, a complete set of neuropsychological assessments is necessary. Future studies need a prospective large sample cohort to quantitatively evaluate the neurotoxic effects of nicotine and non-nicotine compounds in tobacco smoke on human bodies.

## CONCLUSIONS

Urinary nicotine metabolites CotGluc, HyPyBut, OHCotGluc or Cot are mainly associated with attention and calculation or language and praxis, among the elderly in southern China; females and non-smokers are vulnerable populations for cognitive impairment related to environmental tobacco exposure.

## Data Availability

The data supporting this research are available from the authors on reasonable request.
