# Supplementary material for: Urinary nicotine metabolites are associated with cognitive impairment among the elderly in southern China
Source: Tob Induc Dis. 2023 Oct 4;21:123. doi: 10.18332/tid/170423 (PMC10548790; doi:10.18332/tid/170423)
Supplement: Supplementary file 1 [file TID-21-123-s1.pdf]

Urinary nicotine metabolites are associated with cognitive impairment among elderly in southern China

2. Materials and methods

Urinary nicotine and its metabolites concentrations

Urinary concentrations of nicotine and its metabolites, including CotGluc, HyPyBut, OHCotGluc, CNO, NicGluc, NNO, OHCot, Cot were measured by LC-MS/MS (1200 series/6410 Triple Quad LC/MS, Agilent Technologies Inc., Santa Clara, CA, USA) with a minor modification [1]. Briefly, a 500 μL aliquot of urine was mixed with 50 μL of 100 ng/mL internal standard (IS) and 10 μL of 50% formic acid, and the mixture was centrifuged at 12,000 g for 10 min. A SPE column (CNW MCX) was pre-conditioned with 3 mL of pure methanol, 3 mL of ultra-pure water, and 20 mmol/L of ammonium formate buffer (pH=2.5, Shanghai Anpu Experimental Technology Co., Ltd., Shanghai, China). The mixture was added into the pre-conditioned SPE cartridge and then was washed with ammonium formate buffer (20 mmol/L, pH=2.5). The sample was eluted with 1 mL methanol/ammonium hydroxide (90/10, v/v) and dried under the nitrogen condition. The extraction was suspended in 100 μL ultra-pure water. A 10 μL sample was injected into a LC-MS/MS system for analysis of urinary nicotinate and its metabolites. The mobile phase was composed of the solvent A (10 mmol/L ammonium acetate, pH =6.8) and solvent B (solvent A-MeOH) and delivered at a flow rate of 0.5 mL/min.

Chromatographic separation was achieved by altering the mobile phase. The column temperature was kept at 40 °C. The gradient elution procedure was set as follows: The volume ratio of solvents in a binary solvent mixture (solvent A to solvent B) was ranked as follows: 95:6 (0–1 min), 80:20 (2–5 min), 0:100 (6–8 min), 95:5 (8–

11 min); parameters for the ion source and in the positive ion mode were listed below: ion spray voltage: 4.0 kV; ion source heater temperature: 350 °C, atomizing gas pressure: 38 psi, drying gas flow rate: 10 L/min, MS scan mode: positive mode.

As shown in **Table S1** for detection method. The limits of detection (LOD) ranged from 0.04 to 6.60 ng/mL for urinary concentrations of nicotine and its metabolites. Values of urinary nicotine and its metabolites below the LOQ was replaced by LOQ/2.

Reference:

1. Piller M, Gilch G, Scherer G, Scherer M. Simple, fast and sensitive LC-MS/MS analysis for the simultaneous quantification of nicotine and 10 of its major metabolites. *J Chromatogr B Analyt Technol Biomed Life Sci.* **2014**;951-952:7-15.

Table S1

Concentrations of urinary nicotine metabolites among study participants (n = 296).

Urinary nicotine metabolites	LOD (ng/mL)	LOD/2	Median	P5	P25	P75	P95
CotGluc	0.04	0.02	1.07	0.02	0.02	8.12	1099.19
HyPyBut	0.09	0.05	2.47	0.05	0.05	10.01	391.30
OHCotGluc	0.47	0.23	2.80	0.23	0.23	6.35	547.30
CNO	0.12	0.06	0.47	0.06	0.06	1.57	148.74
NicGluc	0.04	0.02	0.26	0.02	0.02	2.11	149.49
NNO	0.09	0.04	0.69	0.04	0.13	2.47	301.08
OHCot	0.56	0.28	2.70	0.28	0.87	8.08	1410.40
Cot	3.55	1.78	0.72	0.04	0.04	3.08	1276.58

Abbreviations: LOD, limit of detection; CotGluc, cotinine N-β-D-glucuronide; HyPyBut, rac 4-hydroxy-4-(3-pyridyl)butanoic acid dicyclohexylamine salt; OHCotGluc, trans-3'-hydroxy cotinine O-β-D-glucuronide; CNO, (S)-cotinine N-oxide; NicGluc, nicotine-N-β-glucuronide; NNO, (1'S, 2'S)-nicotine-1'-oxide; OHCot, trans-3'-hydroxy cotinine; Cot, cotinine.

Table S2

Distribution of cognitive function and urinary nicotine metabolites in gender or smoking status.

**Group**	**Gender**			**Smoking status**		
	Male (n=152)	Female (n=144)	*P*	Smokers (n=72)	Nonsmokers (n=224)	*P*
MMSE total score (mean ± SD)	21.65 ± 3.98	20.69 ± 3.82	0.034	21.21 ± 3.68	21.17 ± 4.01	0.949
Urinary nicotine metabolites (ng/mL, median, IQR)						
CotGluc	2.089 (0.018, 100.003)	0.754 (0.018, 2.614)	<0.001	95.371 (0.018, 522.894)	0.769 (0.018, 3.330)	<0.001
HyPyBut	4.977 (0.047, 37.732)	1.897 (0.047, 5.502)	<0.001	22.562 (1.040, 181.459)	1.912 (0.047, 6.368)	<0.001
OHCotGluc	3.512 (0.230, 19.448)	2.136 (0.616, 5.263)	0.028	13.783 (0.690, 132.868)	2.058 (0.230, 4.998)	<0.001
Cot	1.040 (0.042, 48.013)	0.603 (0.042, 1.364)	0.001	39.672 (0.042, 556.732)	0.604 (0.042, 1.489)	<0.001

Abbreviations: CotGluc, cotinine N-β-D-glucuronide; HyPyBut, rac 4-hydroxy-4-(3-pyridyl)butanoic acid dicyclohexylamine salt; OHCotGluc, trans-3'-hydroxy cotinine O-β-D-glucuronide; Cot, cotinine.

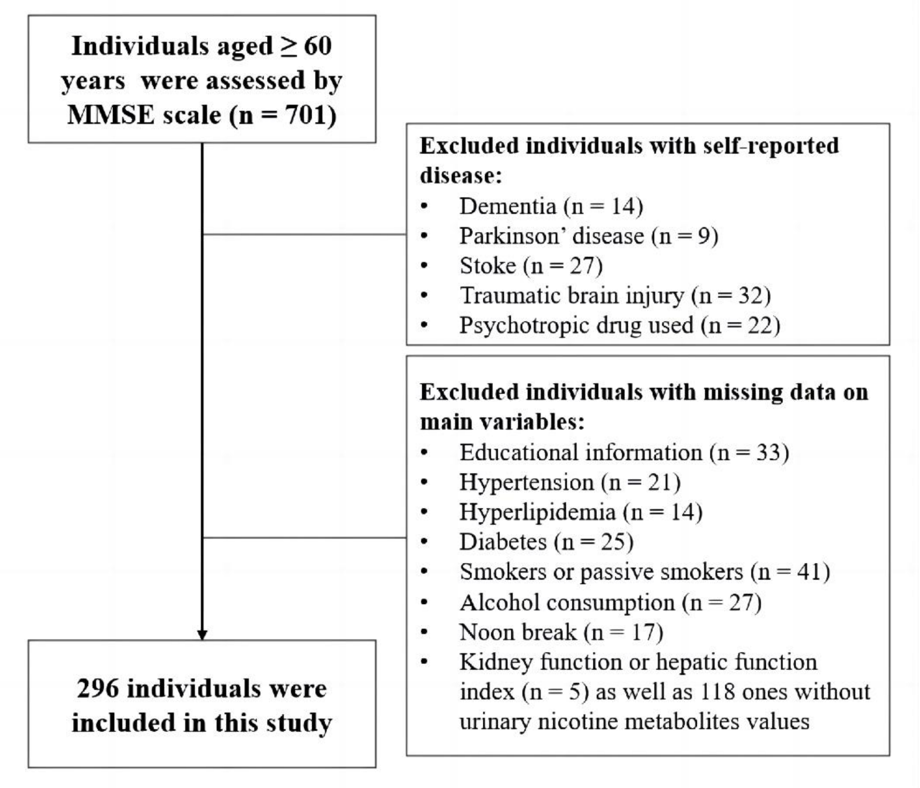

Figure S1. Flowchart of inclusion and exclusion process of participants in the study.

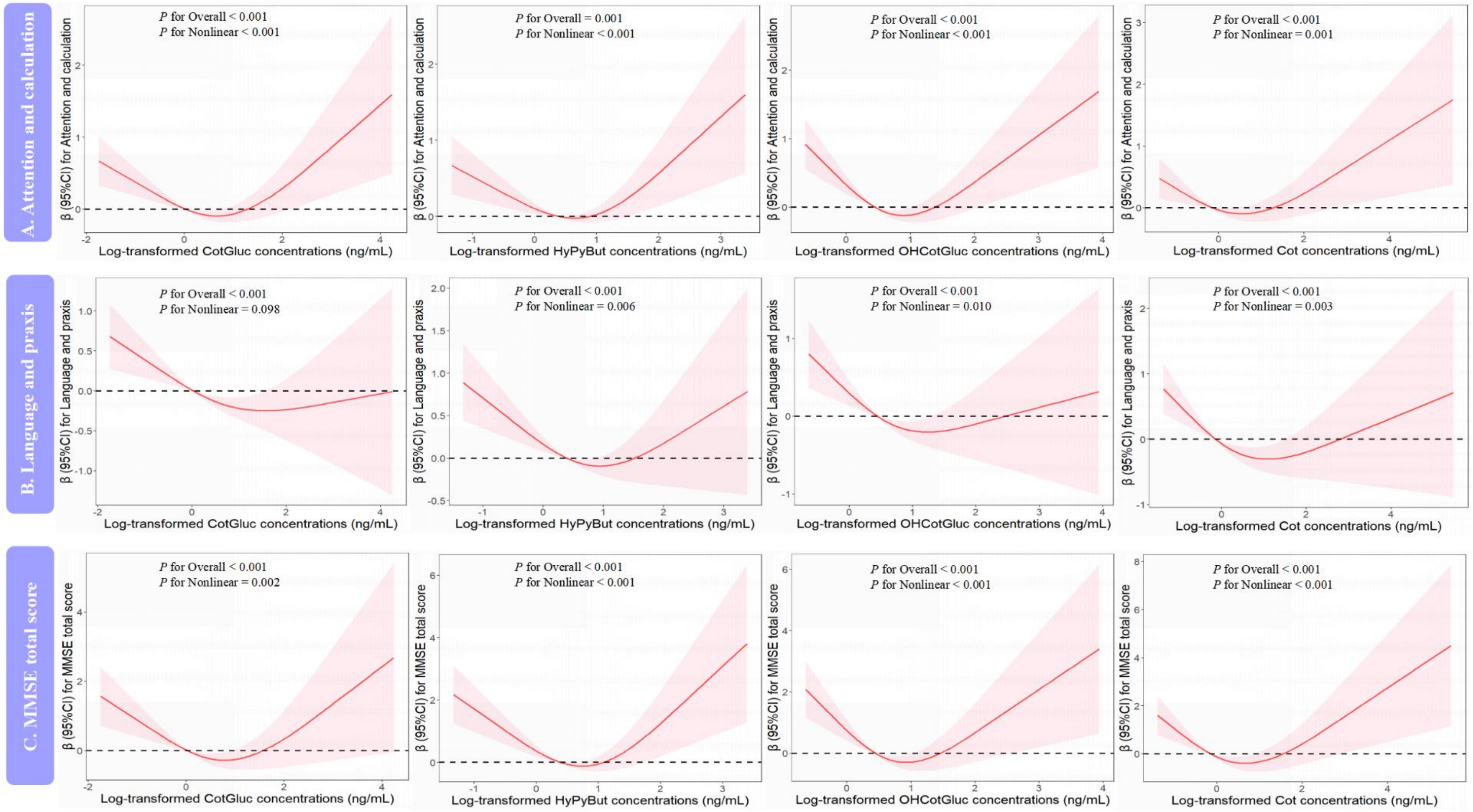

Figure S2. The restricted cubic spline for the association between urinary nicotine metabolites concentrations and attention and calculation (A),

language and praxis (B), and MMSE (C). Knots were placed at the 25th, 50th, and 75th percentiles of the log-transformed urinary nicotine metabolites concentrations, and the corresponding medians of them were set as the reference values. All of the models were adjusted for gender, age, education levels, hypertension, hyperlipidemia, diabetes, active smoking status, passive smoking status, alcohol consumption, noon break, BUN, SUA, SCr, TBill, ALT, AST and urine creatinine. Abbreviations: CotGluc, cotinine N-β-D-glucuronide; HyPyBut, rac 4-hydroxy-4-(3-pyridyl) butanoic acid dicyclohexylamine salt; OHCotGluc, trans-3'-hydroxy cotinine O-β-D-glucuronide; Cot, cotinine.

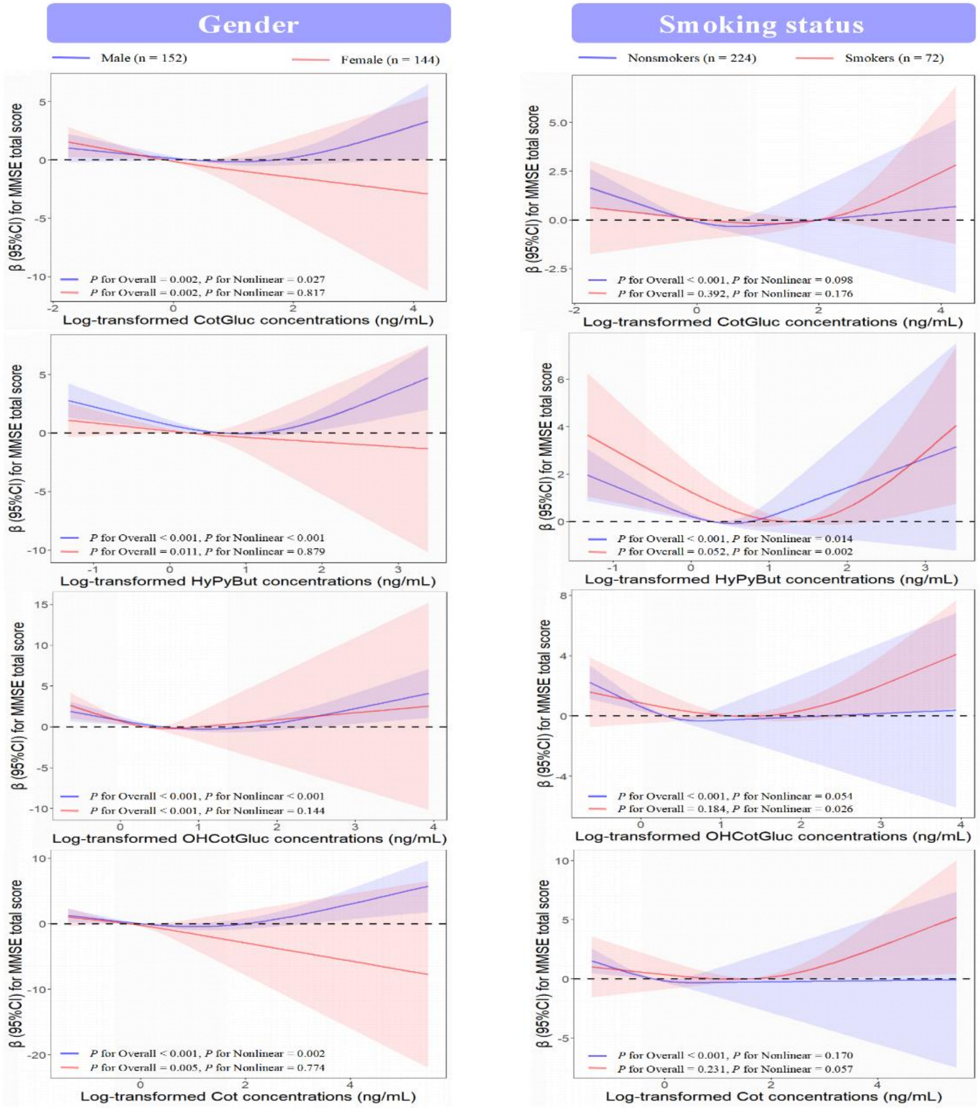

Gender
Smoking status
Male (n = 152)
Female (n = 144)
Nonsmokers (n = 224)
Smokers (n = 72)
β (95%CI) for MMSE total score
P for Overall = 0.002, P for Nonlinear = 0.027
P for Overall = 0.002, P for Nonlinear = 0.817
Log-transformed CotGluc concentrations (ng/mL)
P for Overall < 0.001, P for Nonlinear = 0.098
P for Overall = 0.392, P for Nonlinear = 0.176
P for Overall < 0.001, P for Nonlinear < 0.001
P for Overall = 0.011, P for Nonlinear = 0.879
Log-transformed HyPyBut concentrations (ng/mL)
P for Overall < 0.001, P for Nonlinear = 0.014
P for Overall = 0.052, P for Nonlinear = 0.002
P for Overall < 0.001, P for Nonlinear < 0.001
P for Overall < 0.001, P for Nonlinear = 0.144
Log-transformed OHCotGluc concentrations (ng/mL)
P for Overall < 0.001, P for Nonlinear = 0.054
P for Overall = 0.184, P for Nonlinear = 0.026
P for Overall < 0.001, P for Nonlinear = 0.002
P for Overall = 0.005, P for Nonlinear = 0.774
Log-transformed Cot concentrations (ng/mL)
P for Overall < 0.001, P for Nonlinear = 0.170
P for Overall = 0.231, P for Nonlinear = 0.057

Figure S3. The restricted cubic spline for the association between urinary nicotine metabolites concentrations and cognitive function among gender and smoking status. Knots were placed at the 25th, 50th, and 75th percentiles of the log-transformed urinary nicotine metabolites concentrations, and the corresponding medians of them were set as the reference values. All of the models were adjusted for gender, age, education levels, hypertension, hyperlipidemia, diabetes, active smoking status, passive smoking status, alcohol consumption, noon break, BUN, SUA, SCr, TBill, ALT, AST and urine creatinine. Abbreviations: CotGluc, cotinine N-β-D-glucuronide; HyPyBut, rac 4-hydroxy-4-(3-pyridyl) butanoic acid dicyclohexylamine salt; OHCotGluc, trans-3'-hydroxy cotinine O-β-D-glucuronide; Cot, cotinine.

© 2023 Huang C. et al.
